# Supplementary material for: Drug repurposing for aging research using model organisms
Source: Aging Cell. 2017 Jun 16;16(5):1006–15. doi: 10.1111/acel.12626 (PMC5595691; doi:10.1111/acel.12626)
Supplement: Supplementary file 7 — Data S1 Zip‐Archive of all report cards. [file ACEL-16-1006-s007.zip › RC_LW4.pdf]

LW4

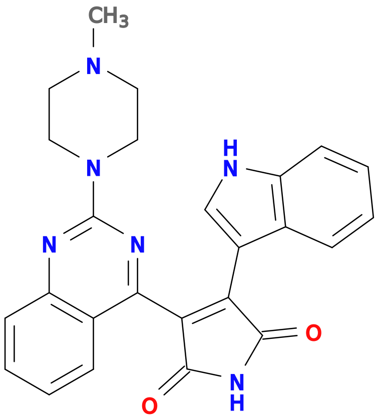

Database identifiers

ChEMBLCompound	CHEMBL565612
eMolecules	36367201

Ranking

	Rank	Score
Drosophila	282/697	0.588
C. elegans	23/591	0.529

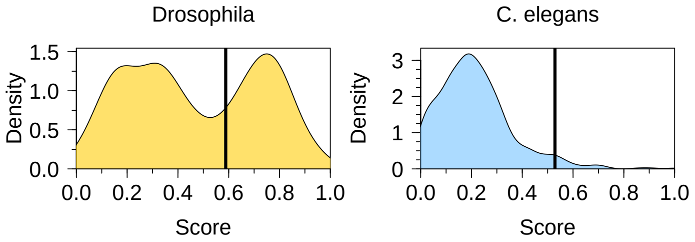

	Ageing implication	Domain conservation	Binding site conservation	Binding affinity	Bioavailability	Lipinski	Promiscuity	Purchasability	Drug approval	**Total**
Drosophila	0.624	0.967	0.951	0.946	(0.9)	0.0	-0.0	0.1	0.0	0.588
C. elegans	0.624	0.96	0.913	0.946	0.829	0.0	-0.0	0.1	0.0	0.529

Names

No synonyms found

Roles

ChEBI entry None has no roles

Status

Approved drug (according to ChEMBL)	No
Number of Rule of 5 violations	0
Binding affinity to original target in log units (RF-Score prediction)	7.86
Burns *C. elegans* bioavailability prediction	6.93

Compound Target Characteristics

Protein kinase C alpha type

Best gene implication in ageing for this target family came from gene F1M2P8 via mapping the annotation from RGD 3395 annotated in RGD 2014-03-11. Annotation GO 7568 (aging) was Inferred from Expression Pattern

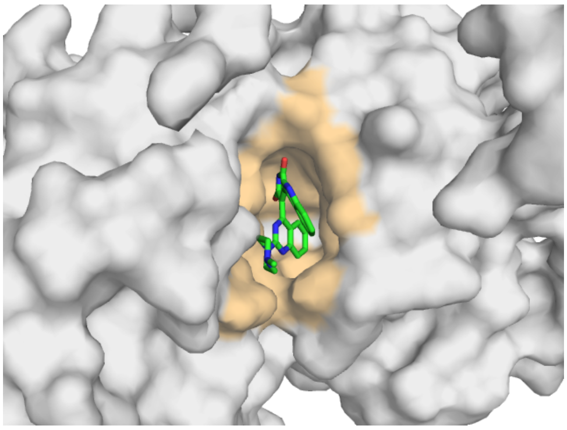

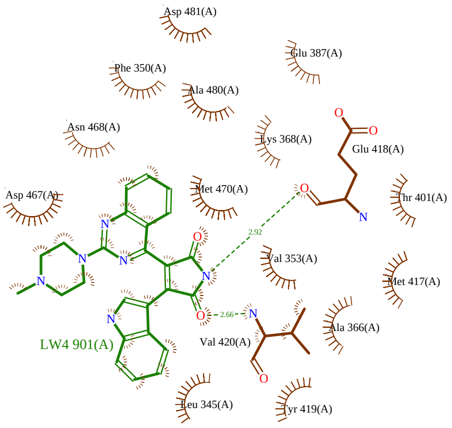

protein	amino acids contacts (binding site)															
PDB:3iw4:chainA:P17252	L	F	V	A	K	E	T	M	E	Y	V	D	N	M	A	D
tr:L7RSM7:L7RSM7_HUMAN	L	F	V	A	K	E	T	M	E	Y	V	D	N	M	A	D
sp:P17252:KPCA_HUMAN	L	F	V	A	K	E	T	M	E	Y	V	D	N	M	A	D
tr:F1M2P8:F1M2P8_RAT	L	F	V	A	K	E	T	M	E	Y	V	D	N	M	A	D
tr:F1LS98:F1LS98_RAT	L	F	V	A	K	E	T	M	E	Y	V	D	N	M	A	D
tr:Q4VA93:Q4VA93_MOUSE	L	F	V	A	K	E	T	M	E	Y	V	D	N	M	A	D
tr:D3DMK5:D3DMK5_DROME	L	F	V	A	K	E	V	M	E	Y	V	D	N	L	A	D
tr:A8DYG9:A8DYG9_DROME	L	F	V	A	K	E	V	M	E	Y	V	D	N	L	A	D
sp:P05130:KPC1_DROME	L	F	V	A	K	E	V	M	E	Y	V	D	N	L	A	D
sp:P90980:KPC2_CAEEL	L	F	V	A	K	E	V	M	E	F	V	D	N	M	T	D
tr:G8JY36:G8JY36_CAEEL	L	F	V	A	K	E	V	M	E	F	V	D	N	M	T	D

	whole protein		domain-based		contact-based	
protein	ident	simil	ident	simil	ident	simil
PDB:3iw4:chainA:P17252	0.99	1.0	0.99	1.0	1.0	1.0
tr:L7RSM7:L7RSM7_HUMAN	1.0	1.0	1.0	1.0	1.0	1.0
sp:P17252:KPCA_HUMAN	1.0	1.0	1.0	1.0	1.0	1.0
tr:F1M2P8:F1M2P8_RAT	0.92	0.94	0.83	0.86	1.0	1.0
tr:F1LS98:F1LS98_RAT	0.97	0.99	0.95	0.98	1.0	1.0
tr:Q4VA93:Q4VA93_MOUSE	0.99	1.0	1.0	1.0	1.0	1.0
tr:D3DMK5:D3DMK5_DROME	0.54	0.71	0.78	0.94	0.88	0.95
tr:A8DYG9:A8DYG9_DROME	0.64	0.86	0.78	0.94	0.88	0.95
sp:P05130:KPC1_DROME	0.65	0.86	0.78	0.94	0.88	0.95
sp:P90980:KPC2_CAEEL	0.66	0.87	0.74	0.92	0.81	0.91
tr:G8JY36:G8JY36_CAEEL	0.62	0.83	0.74	0.92	0.81	0.91

Pkc53E (FBgn0003091) associated phenotypes

dominant, size defective

(Information from FlyBase)

Pkc53E (UniProt:P05130) annotation

Function: PKC is activated by diacylglycerol which in turn phosphorylates a range of cellular proteins. PKC also serves as the receptor for phorbol esters, a class of tumor promoters.
Tissue specificity: Head neural tissue.
Miscellaneous: This is a calcium-activated, phospholipid- dependent, serine- and threonine-specific enzyme.
Miscellaneous: The sequence shown is that of Oregon-R.

(Information from UniProt)

pkc-2 (WBGene00004033) associated phenotypes
shortened life span

(Information from WormBase)

pkc-2 (UniProt:P90980) annotation
Function: PKC is activated by diacylglycerol which in turn phosphorylates a range of cellular proteins. PKC also serves as the receptor for phorbol esters, a class of tumor promoters.

(Information from UniProt)
